# Treatment of Naïve Patients with Chronic Hepatitis C Genotypes 2 and 3 with Pegylated Interferon Alpha and Ribavirin in a Real World Setting: Relevance for the New Era of DAA

**DOI:** 10.1371/journal.pone.0108751

**Published:** 2014-10-10

**Authors:** Benjamin Heidrich, Steffen B. Wiegand, Peter Buggisch, Holger Hinrichsen, Ralph Link, Bernd Möller, Klaus H. W. Böker, Gerlinde Teuber, Hartwig Klinker, Elmar Zehnter, Uwe Naumann, Heiner W. Busch, Benjamin Maasoumy, Undine Baum, Svenja Hardtke, Michael P. Manns, Heiner Wedemeyer, Jörg Petersen, Markus Cornberg, for the HepNet Study Group

**Affiliations:** 1 Department of Gastroenterology, Hepatology and Endocrinology, Hannover Medical School, Hannover, Germany; 2 German Liver Foundation, HepNet Study-House, Hannover, Germany, and German Center for Infection Research (DZIF), Partner Site Hannover-Braunschweig, Braunschweig, Germany; 3 IFI Institute for Interdisciplinary Medicine, Asklepios Klinik St Georg, Hamburg, Germany; 4 Gastroenterology, Gastroenterologische Schwerpunkt Praxis, Kiel, Germany; 5 St. Josefs hospital, Offenburg, Germany; 6 Medical Practice, Berlin, Germany; 7 Leberpraxis, Hannover, Germany; 8 Gastroenterological Practice, Frankfurt, Germany; 9 Dept. of Internal Medicine II, University of Würzburg, Würzburg, Germany; 10 Gastroenterological Practice, Dortmund, Germany; 11 Center for Addiction-Medicine, Hepatology and HIV, Praxiszentrum Kaiserdamm, Berlin, Germany; 12 Medical Practice, Münster, Germany; University of Sydney, Australia

## Abstract

Evidence based clinical guidelines are implemented to treat patients efficiently that include efficacy, tolerability but also health economic considerations. This is of particular relevance to the new direct acting antiviral agents that have revolutionized treatment of chronic hepatitis C. For hepatitis C genotypes 2/3 interferon free treatment is already available with sofosbuvir plus ribavirin. However, treatment with sofosbuvir-based regimens is 10–20 times more expensive compared to pegylated interferon alfa and ribavirin (PegIFN/RBV). It has to be discussed if PegIFN/RBV is still an option for easy to treat patients. We assessed the treatment of patients with chronic hepatitis C genotypes 2/3 with PegIFN/RBV in a real world setting according to the latest German guidelines. Overall, 1006 patients were recruited into a prospective patient registry with 959 having started treatment. The intention-to-treat analysis showed poor SVR (GT2 61%, GT3 47%) while patients with adherence had excellent SVR in the per protocol analysis (GT2 96%, GT3 90%). According to guidelines, 283 patients were candidates for shorter treatment duration, namely a treatment of 16 weeks (baseline HCV-RNA <800.000 IU/mL, no cirrhosis and RVR). However, 65% of these easy to treat patients have been treated longer than recommended that resulted in higher costs but not higher SVR rates. In conclusion, treatment with PegIFN/RBV in a real world setting can be highly effective yet similar effective than PegIFN± sofosbuvir/RBV in well-selected naïve G2/3 patients. Full adherence to guidelines could be further improved, because it would be important in the new era with DAA, especially to safe resources.

## Introduction

More than 150 million people world-wide and 8–11 million people in Europe are chronically infected with the hepatitis C virus (HCV) [1], [2]. Patients with chronic hepatitis C are at risk to develop liver cirrhosis and hepatocellular carcinoma [3]. During the last 15 years there has been an enormous achievement in the diagnosis, management, and therapy of hepatitis C. Analysis of HCV-genotypes (GT), quantification of HCV-RNA viral load, and calculation of viral kinetics allow better management of patients with chronic hepatitis C. The standard treatment until recently consisted of pegylated interferon alpha (PegIFN) and ribavirin (RBV) [4]. Since 2011, the first direct acting antiviral agents (DAA) have been approved. The first generation protease inhibitors boceprevir and telaprevir were only approved for genotype 1 and combination with PegIFN and RBV was still necessary because monotherapy resulted in rapid emergence of drug resistance [5]. However, the availability of further DAA has already revolutionized the treatment of chronic hepatitis C. The main targets for DAA are the NS3/4A protease, NS5B polymerase and the NS5A replication complex. Combinations of different DAA from different classes will allow very potent treatments even without PegIFN [6]. In particular, therapy of GT2/3 has changed in 2014 with the approval of sofosbuvir (SOF). SOF is a new NS5B polymerase inhibitor with pangenotypic efficacy and extensive data were acquired in the treatment of GT2- and GT3-infected patients, which were the basis for the approval for the first interferon-free treatment of hepatitis C [7]–[9]. However, treatment with PegIFN/RBV dual therapy may be still considered depending on the health care system, especially for easy-to-treat GT2/3 patients. Treatment with SOF/RBV therapy for 12 to 24 weeks or SOF in combination PegIFN and RBV in HCV genotype 2 or 3 can be 10–20 times more expensive compared to PegIFN and RBV treatment [10].

For Peg-IFN/RBV a fixed duration of treatment (24 weeks) has been suggested [11], although the optimal results are likely to be achieved when the duration of therapy is adjusted based on viral kinetics. Many studies have investigated the reduction of treatment duration for HCV GT2/3 to 16, 14, or even 12 weeks [12]–[14]. Overall, reducing the treatment duration to less than 24 weeks increases the number of relapses. However, some HCV GT2/3 patients may indeed be treatable for 12–16 weeks if certain prerequisites are fulfilled, especially the rapid virologic response (RVR) by week 4 of therapy [15]. In addition to the RVR, the specific HCV genotype and the baseline viral load are associated with response [12]. Patients with low baseline viral load <800.000 IU/ml and RVR have high SVR rates>85% after 16 weeks, 14 weeks, or even 12 weeks of therapy. Reducing treatment duration is not recommended for patients with advanced liver fibrosis or cirrhosis, insulin resistance, diabetes mellitus or BMI>30 kg/m^2^ [15]. Thus, recent clinical guidelines recommended that naïve patients with GT2/3 plus low viral load who achieve RVR can be treated shortly, i.e. 16 weeks according to the German Guidelines [15].

A major aim of this study was to find out if patients with GT2/3 were treated according to guidelines and if this treatment is efficient. We also discussed the results in the context of the new SOF-based treatment for GT2/3.

## Materials and Methods

### Patient population

The Competence Network for Viral Hepatitis in Germany (HepNet) implemented a nationwide multicenter prospective registry for naïve patients chronically infected with hepatitis C virus (HCV) genotype 2 and 3. MSD Merck Sharp & Dohme sponsored the registry (financial sponsorship only). The inclusion criteria (File S1) allowed treatment with both pegylated interferons (PegIFN a2a, PegIFN a2b).

**Figure 1:**
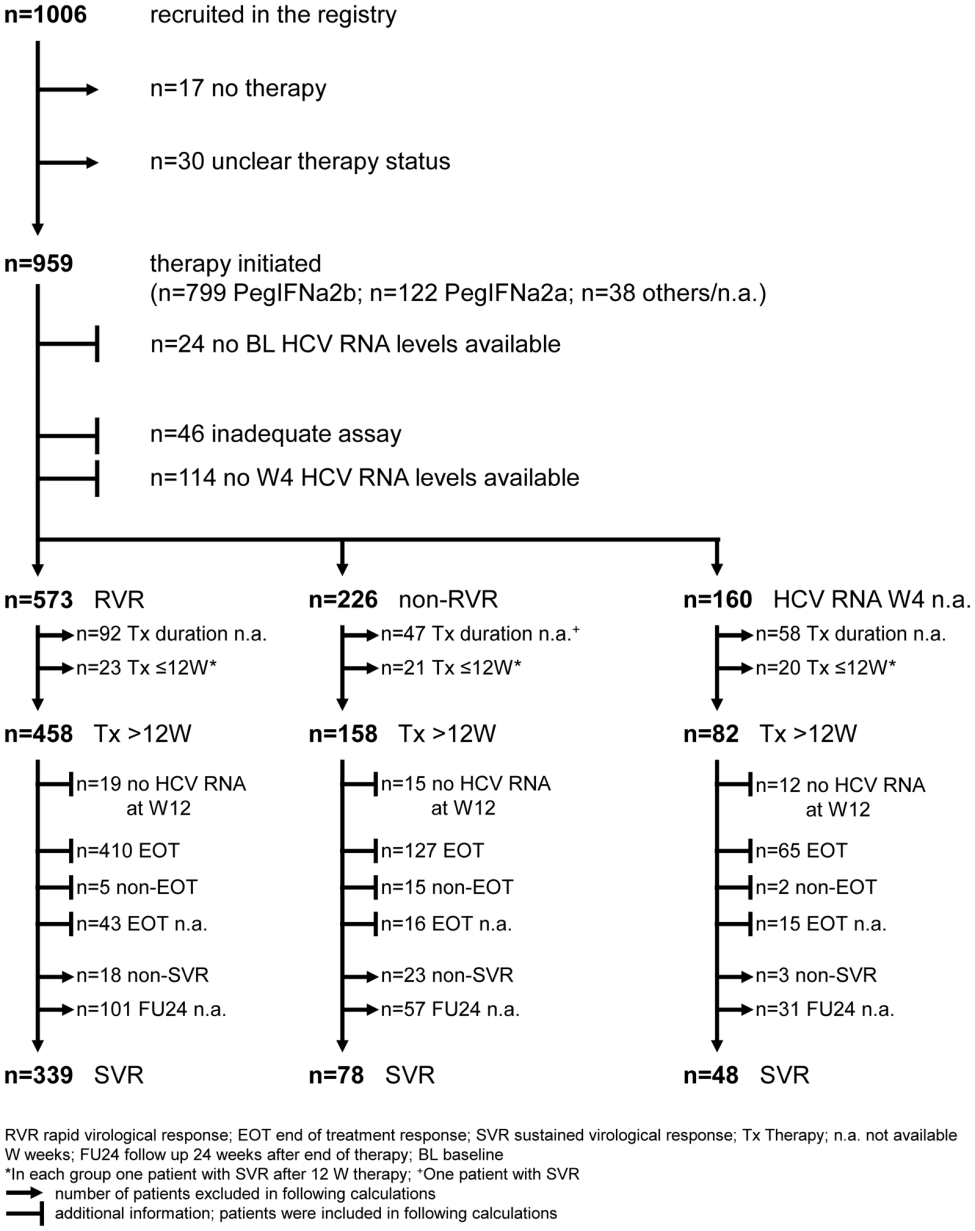
Flow chart of patients recruited in the HCV genotype 2/3 registry.

Between June 2008 and December 2012 a total of 1006 patients were recruited in 72 centers in Germany. All patients at the age of 18 or older with chronic hepatitis C genotype 2 or 3 infection, detectable plasma HCV RNA and positivity of anti-HCV antibodies as well as no history of antiviral therapy were eligible (Figure 1).

### HCV RNA quantification

HCV RNA was assessed at baseline, week 4, −12, end of treatment and 24 weeks after cessation of therapy. Samples were tested locally with different assays and thresholds. The Cobas-TaqMan assay with a lower limit of quantification (LoQ) of 15 IU/mL was used in 57%, the Abbott RealTime assay in 8% with a LoQ of 12 IU/mL and the Roche Amplicor assay with LoQ of 50 IU/mL in 5%. Additionally, in 24% the used assay was not indicated and in 7% others or in-house assays with different cut-offs were used. Assays were used according to manufacturer's instructions.

### Laboratory results

Several Biochemical and hematological parameters at baseline were assessed locally. Biochemical markers included alanine aminotransferase (ALT), aspartate aminotransferase (AST), gamma-glutamyltransferase (GGT) and alkaline phosphatase (ALP) as well as bilirubin, creatinine and albumin. Hematological parameters included platelet counts and prothrombin time.

### Definitions of response to therapy

Rapid virological response (RVR) was defined as HCV RNA below 15 IU/mL at week 4 of treatment with pegylated interferon alpha (PegIFN) and ribavirin (RBV), early virological response (EVR) was defined as ≥2 log10 decrease from baseline in HCV RNA or HCV RNA negativity at week 12; end of treatment response (EOT) and sustained virological (SVR) were defined as HCV RNA below detection limit at the end of treatment and 24 weeks after the end of treatment, respectively [15]. In the intention-to-treat (ITT) analysis all patients with at least one dose of PegIFN and RBV were included. Missing results for RVR and SVR were considered as negative results. For the per-protocol (PP) analysis, patients treated for at least 12 weeks and with available results at week 4 and/or 24 weeks after end of therapy were considered for each evaluation.

### Definition of liver cirrhosis

Diagnosis of cirrhosis was either based on liver histology or non-invasive methods such as ultrasound, FibroScan or biochemical results. Liver cirrhosis in biopsies was defined as F4 in Metavir or F5-6 in ISHAK. In addition, diagnosis of liver cirrhosis was based on ultrasound results assessed by the local physician. Liver stiffness ≥12.5 kPa was considered as cirrhosis [16]. Patients with at least two of the following criteria platelets <100/nL, AST/ALT ratio>1, bilirubin>1.5 ULN and albumin <35 g/L fulfilled biochemical assessment of cirrhosis. Individuals were considered having cirrhosis if one of the definitions above was imbued.

### Statistical analysis

For statistical analysis as well as for graphic design we used SPSS, version 15.0.1 (November 2006, SPSS, Munich, Germany) and GraphPad Prism 5 (GraphPad Software, Inc., La Jolla, CA, USA). Quantitative values are indicated in median and statistical differences were assessed by using Student t test. In case of analysis of qualitative data we used Chi square test. Differences between clinical outcomes were determined using Cox regression analysis. Differences were considered significant at p≤0.05.

### Ethical approval

Ethics committee at each participating institution approved the non-interventional patient registry of the German Competence Network for Viral Hepatitis (Hep-Net) and each patient signed a written informed consent form. The registry has been performed according to the World Medical Association Declaration of Helsinki (http://www.wma.net/e/policy/b3.htm). The procedures have been approved by the local ethics committee of the Hannover Medical School (Vote No. 3860) and are in line with German law.

## Results

Between June 2008 and December 2012 a total of 1006 patients were included. 959 patients received at least one dose of pegylated interferon alfa (PegIFN) and ribavirin (RBV). Two out of three patients were male with a median age of 43.8 years and BMI of 25.0 kg/m^2^. The majority of patients originated from Germany and was infected with genotype (GT) 3. Hepatic steatosis was present in about one-third of patients and cirrhosis was diagnosed in 8.0% (Table 1, 2, and 3).

**Table 1:** Baseline characteristics of all patients who started treatment with PegIFN/RBV.

Parameter*	Value	Range	n =
**Male [%]**	65.7		n = 617/939
**Age [years]**	43.8±10.6	20.1–81.8	n = 957
**Weight [kg]**	76.0±16.9	40.0–162.0	n = 931
**BMI [kg/m^2^]**	25.0±4.8	15.2–57.7	n = 919
**CoO Germany [%]**	60.0		n = 538
**CoO EE/USSR [%]**	30.8		n = 276
**CoO Mediterranen [%]**	6.1		n = 55
**CoO Others [%]**	3.0		n = 27
**Genotype 3 [%]**	82.7		n = 769
**HCV RNA [log10 IU/mL]**	5.8±1.0	1.2–8.7	n = 934
**ALT [U/L]**	89.0±107.7	11.0–1273.0	n = 909
**Cirrhosis [%]**	7.8		n = 75/959
**Steatosis [%]**	28.8		n = 225/782

**Table 2:** Baseline characteristics of patients with HCV Genotype 2 who started treatment with PegIFN/RBV.

Parameter*	Value	Range	n =
**Male [%]**	63.4		n = 102/161
**Age [years]**	49.8±12.0	26.4–79.2	n = 160
**Weight [kg]**	78.0±16.4	47.0–149.0	n = 160
**BMI [kg/m^2^]**	26.1±4.9	16.7–45.0	n = 158
**CoO Germany [%]**	53.1		n = 85
**CoO EE/USSR [%]**	34.4		n = 55
**CoO Mediterranen [%]**	8.8		n = 14
**CoO Others [%]**	3.8		n = 6
**HCV RNA [log10 IU/mL]**	6.2±1.1	2.7–8.2	n = 154
**ALT [U/L]**	68.0±93.0	14.0–542.0	n = 152
**Cirrhosis [%]**	5.6		n = 9/161
**Steatosis [%]**	29.3		n = 41/140

**Table 3:** Baseline characteristics of patients with HCV Genotype 3 who started treatment with PegIFN/RBV.

Parameter*	Value	Range	n =	p#
**Male [%]**	66.0		n = 506/767	0.5250
**Age [years]**	42.2±9.9	20.1–81.8	n = 761	<0.0001
**Weight [kg]**	75.0±16.9	40.0–162.0	n = 760	0.0049
**BMI [kg/m^2^]**	24.6±4.7	15.2–57.7	n = 751	<0.0001
**CoO Germany [%]**	61.2		n = 447	0.0580
**CoO EE/USSR [%]**	30.5		n = 223	0.3440
**CoO Mediterranen [%]**	5.5		n = 40	0.1170
**CoO Others [%]**	2.7		n = 20	0.4920
**HCV RNA [log10 IU/mL]**	5.8±1.0	1.2–8.7	n = 753	0.0007
**ALT [U/L]**	93.0±109.4	11.0–1273.0	n = 745	0.0018
**Cirrhosis [%]**	8.6		n = 66/769	0.2050
**Steatosis [%]**	28.6		n = 182/636	0.8740

*Continuous values are indicated in median.

# Chi-Square or t-test results.

CoO = Country of Origin.

The Majority of patients received PegIFN-2b (n = 799; 83%) whereas 122 patients were treated with PegIFN-2a. For the remaining 38 patients the exact treatment regimen was not fully specified. In 24 patients (3%) baseline HCV RNA levels were not determined (Figure 1).

### HCV RNA assays used for diagnosis and monitoring

Since our cohort consists of patients treated within a prospective registry in a real world setting in multiple centers several different HCV RNA assays were used. Overall, in the far majority of cases the used assays were indicated (n = 765; 76%). Importantly, in 46 cases out of 765 (6%) in-house PCRs were used instead of commercially available assays. Most of the patients (n = 691; 90%) were monitored with very sensitive assays with limits of quantification of 15 IU/mL. However, in 62 (8%) patients assays with moderate sensitivity (LoQ 50 IU/mL) and in 8 (1%) patients assays with low sensitivity (LoQ>50 IU/mL) were used.

### Efficacy of treatment

**Figure 2:**
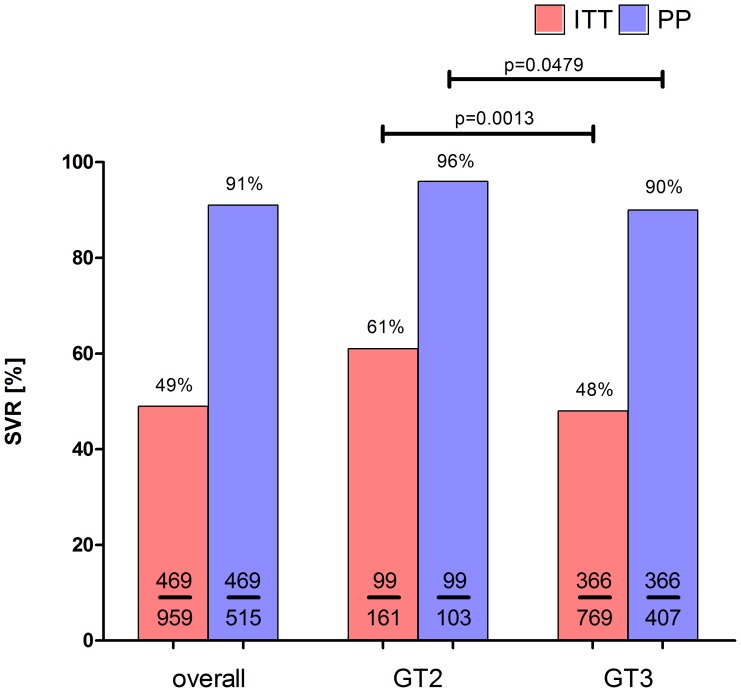
SVR rates in all patients with genotypes 2 and 3 (ITT and PP analysis).

The overall sustained virological response (SVR) was 49% in the intention-to-treat (ITT) analysis and 91% in the per-protocol (PP) analysis (Figure 2). Individuals with HCV GT2 had significantly higher SVR rates than GT3 patients in the ITT and PP analysis, respectively (ITT 61% vs. 48%; p = 0.0013; PP 96% vs. 90%; p = 0.0479) (Figure 2).

Rapid virological response (RVR) was achieved by 573 of all treated patients while 226 had HCV RNA levels above 15 IU/mL four weeks after therapy initiation. In the remaining 160 individuals HCV RNA quantification was not performed at week 4 or the sensitivity of the used assays was inadequate to define RVR (Figure 1). Thus, RVR was achieved in 573/799 (72%). In patients with GT3, 70% achieved RVR, while this happened more often in GT2 patients (70% vs. 78%; p = 0.052).

**Figure 3:**
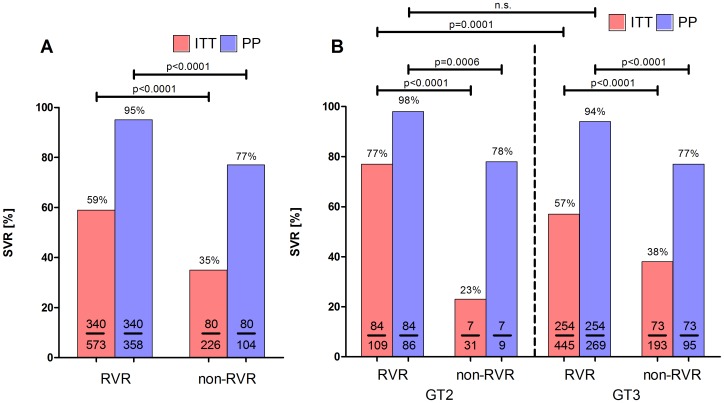
SVR rates according to HCV genotype and week 4 response. A: SVR rates in patients with or without RVR. B: SVR rates in patients with GT2 or 3 with or without RVR.

In the RVR group 340 patients achieved SVR, which is significantly higher compared to patients without RVR (ITT: 59% vs. 35% and PP: 95% vs. 77%; p<0.0001 for both) (Figure 3A). RVR was a predictor of SVR irrespective from the present GT 2 or 3 (Figure 3B). Individuals without or inadequate HCV RNA testing at week 4 achieved SVR rates of 31% and 92% in ITT and PP analysis, respectively. Patients with RVR were significantly younger (42.1 vs. 48.3 [years]; p<0.0001), had lower HCV RNA levels (5.8 vs. 6.1 [log 10 IU/mL]; p<0.0001) at baseline and had less often signs of cirrhosis (6% vs. 11%; p = 0.009) compared to patients without RVR (Table 4). Interestingly, no differences in the frequency of steatosis, dose of PegIFN and RBV, respectively, were observed (Table 4).

**Table 4:** Baseline characteristics of patients with RVR and non-RVR.

Parameter*	RVR+ n = 573			non-RVR+ n = 226			p-value
	Value	Range	n =	Value	Range	n =	
**Male [%]**	65		n = 365/562	68		n = 151/223	0.4614
**Age [years]**	42.1±11.1	2.5–81.8	n = 560	48.3±9.7	20.1–66.4	n = 221	<0.0001
**Weight [kg]**	76.3±16.9	40.0–150.0	n = 557	76,0±16.3	47.0–140.0	n = 224	0.5197
**BMI**	25.2±4.8	15.2–51.2	n = 551	25.0±4.5	16.7–45.9	n = 221	0.5462
**CoO Germany [%]** ++	58		n = 311/540	63		n = 142/214	0.1374
**Genotype 3 [%]**	80		n = 445/554	86%		n = 193/224	0.0550
**HCV RNA [log 10 IU/mL]**	5.8±1.0	1.9–8.7	n = 563	6.1±0.8	2.7–7.5	n = 221	<0.0001
**ALT [U/L]**	2.3±2.5	0.3–27.8	n = 543	2.0±2.3	0.2–12.5	n = 219	0.5226
**Cirrhosis [%]**	6		n = 33/573	11		n = 25/226	0.0093
**Steatosis [%]**	29		n = 136/470	33		n = 60/184	0.3566
**Dose of IFN** ** **[µg/kg BW** # **]**	1.5±0.7	0.5–13.8	n = 543	1.5±0.4	1.0–3.3	n = 221	0.4437
**Dose of RBV** ∼ **[mg/kg BW** # **]**	13.3±2.0	4.2–22.7	n = 539	13.5±2.2	1.8–28.6	n = 216	0.7976

*Continuous values are indicated in median.

+ Not all parameters are available for each patient. Only available parameter were considered for calculations.

++ Country of Origin.

**Interferon.

∼ Ribavirin.

# body weight.

### Compliance to the current German clinical guideline

According to the German clinical practice guideline patients chronically infected with hepatitis C virus genotype 2/3 should in principle be treated for 24 weeks. However, in naïve patients with low viral load (<800.000 IU/mL and no signs of cirrhosis) response-guided treatment (RGT) is strongly recommended. If these patients achieve RVR, a shortening of the treatment duration to 16 weeks is proposed with a grade A recommendation [15]. Patients with HCV RNA decline less than 2log10 after 12 weeks of therapy (no EVR) should be stopped immediately [15].

**Figure 4:**
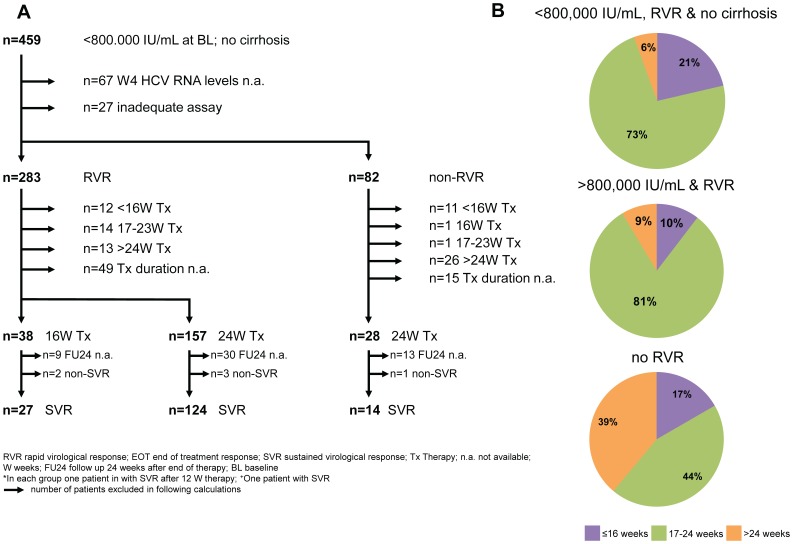
Therapy regimens and treatment durations in different groups of patients. A: Patients are divided in three different groups according to week 4 response and duration of therapy. B: The first group consists of the so called easy to treat patients with low viral load, no cirrhosis and RVR. In the second group are patients with RVR but high viral load irrespective of stage of liver fibrosis and the last group contains all patients with non-RVR.

**Figure 5:**
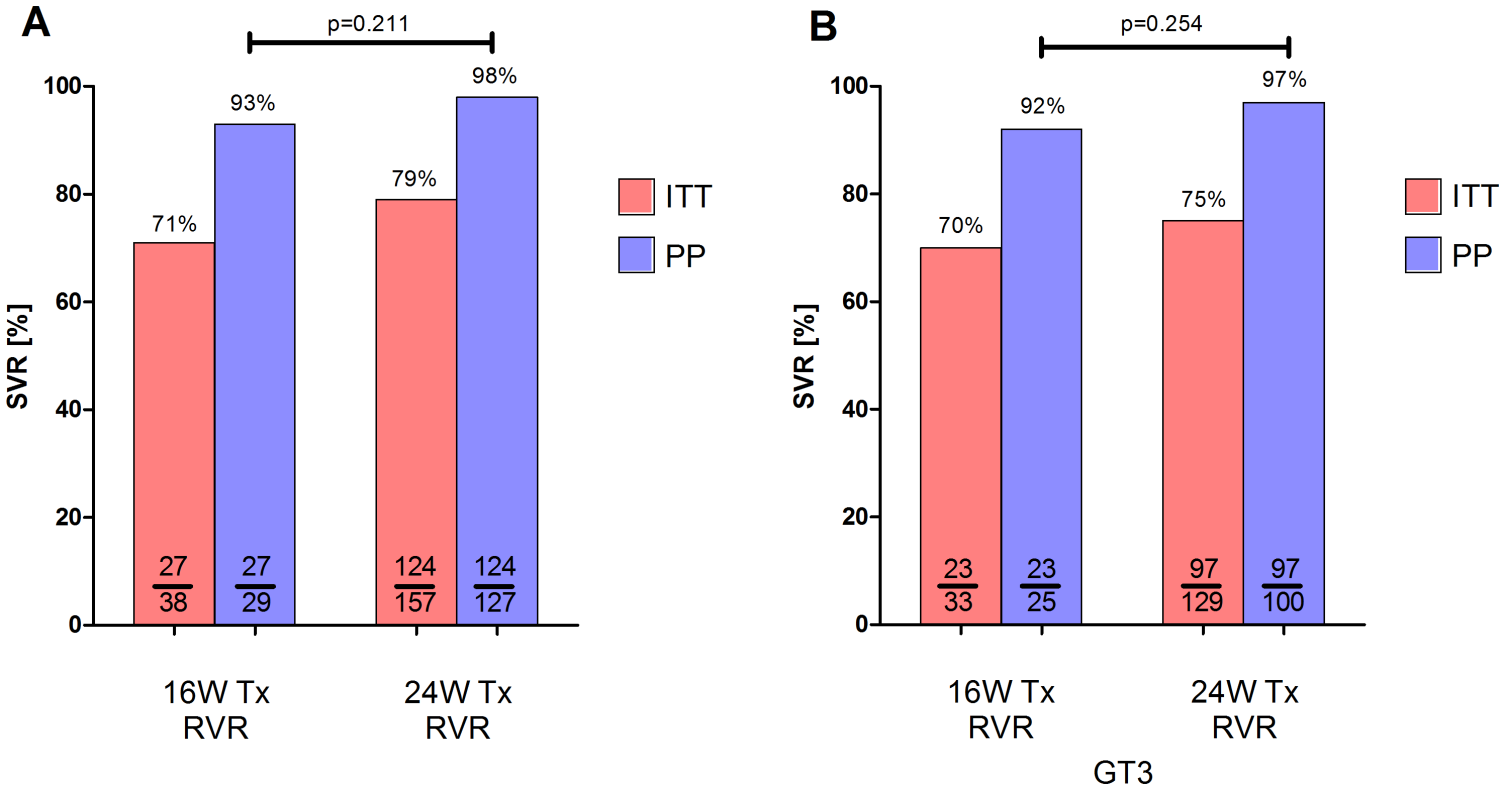
SVR rates in patients with RVR treated for 16 or 24 weeks in the overall cohort and in HCV genotype 3 patients. A: All patients had low viral, no signs of cirrhosis, RVR and were recommended for 16 weeks of treatment. B: All patients had low viral, no signs of cirrhosis, RVR and were recommended for 16 weeks of treatment.

In our cohort, 459 patients fulfilled baseline criteria for RGT with PegIFN and RBV (Figure 4A). In 24 (3%) individuals HCV RNA testing was not performed at baseline. Out of 459 patients, 283 (62%) individuals achieved RVR, while 82 (18%) had HCV RNA levels above 15 IU/mL. Importantly, in 67 (15%) patients no HCV RNA quantification at week 4 was done. In 27 (6%) patients assays with low sensitivity were used, thus therapy shortening could not be decided. Overall, 283 patients treated in 48 centers fulfilled the criteria for RGT according to the German guideline. Interestingly, only 38 (13%) patients were treated for 16 weeks. In the majority of cases the physicians did not follow the recommended RGT criteria and patients were treated for 24 weeks (n = 157; 55%) (Figure 4B). Out of the 48 centers, there were 9 centers with only one patient applicable for RGT. Therapy shortening was done in 2 out of 9 (22%) individuals. Out of 39 centers with at least 2 patients with option for shortening to 16 weeks only one center treated its patients for 16 weeks. Only 9 (23%) centers considered RGT in at least some of their patients. Thirty-three out of 39 (85%) centers treated their patients predominantly for 24 weeks or even longer up to 48 weeks. Importantly, the SVR rates in the 16-week as well as in the 24-week cohort were almost similar to more than 90% in the PP analysis (ITT: 71% vs. 79%; p = 0.294 and PP: 93% vs. 98%; p = 0.211) (Figure 5A). The same was true for GT3 (Figure 5B).

In addition, we analyzed non-cirrhotic patients with low baseline viral load who did not achieve RVR. Overall, 82 patients with low viral load and no signs of cirrhosis at baseline did not achieve RVR. According to the German guideline these patients may even be considered for longer treatment based on limited evidence [15]. In this registry 28 out of 82 (34%) were treated for 24 weeks (Figure 4A). About one third of patients (n = 26; 32%) received therapy longer than 24 weeks including 10 individuals treated for 48 weeks. For 15 patients treatment duration was not assessed. Importantly, the SVR rate was again above 90% in patients treated for 24 weeks and was comparable to all patients with RVR (data not shown). Therapy prolongation longer than 24 weeks did not lead to higher SVR rates in this registry with a relatively low number of patients (Tx 24 weeks 93% vs. Tx>24 weeks 82%; p = 0.3144). All patients, receiving therapy for more than 12 weeks, achieved EVR. Of note, 46 (7%) patients were not tested for HCV RNA at week 12.

Also patients with RVR and high viral load at baseline and/or liver cirrhosis had SVR rate of 94% and were comparable to patients with low viral load and no signs of cirrhosis (94% vs. 93%; p = 0.773).

Patients, who were treated with PEGIFN/RBV very efficacy, were patients with cirrhosis and non-RVR. In our registry only 20% (5/25) in the ITT and 50% (5/10) in the PP analysis achieved SVR. Median treatment duration was 24 weeks.

### Additional costs due to over-treatment

Within our cohort many patients were treated longer than recommended in the German guideline due to different reasons, thus additional costs were generated. The majority of patients was treated with PegIFN-2b and had a mean weight of 77.4 kg. Based on these findings the approximate therapy costs for four weeks with 120 µg Peg-IFN-2b (PEGINTRON, MSD Whitehouse Station, NJ, USA) plus RBV (REBETOL, MSD Whitehouse Station, NJ, USA) are 2,346 €. In total 210 patients with low viral load and no signs of cirrhosis were treated longer than recommended (mean 22 weeks, median 24 weeks), which lead to additional costs of 897,589 € and 20 individuals with high viral load and/or signs of cirrhosis but with RVR were treated longer than 24 weeks (148,637 €) in contrast to the German guideline. Consequently over-treatment of patients with HCV GT2/3 resulted in further costs of 1,046,222 € in our cohort, 4,549 € per patient who was treated longer than recommended in agreement with the German guideline. Of note, 26 patients with neither high viral load nor signs of cirrhosis but no RVR were treated longer than 24 weeks, which is based on limited evidence, resulting in extra costs of 238,344 €.

## Discussion

Treatment of chronic hepatitis C is currently an area of dramatic changes. Since the approval of sofosbuvir (SOF), interferon free treatment is already available for genotypes 2 and 3 patients and suggested as standard of care by the new AASLD [17] and EASL guidelines [18]. However, this treatment revolution may be not available in all areas of the world at the same time and even if sofosbuvir is available everywhere, treatment may be not affordable for some health care systems. Thus it has to be discussed if the old standard of care, PegIFN and RBV may be considered for certain patients.

Our prospective patient registry revealed several very important findings: First, in a large, representative real-life cohort with more than 150 involved centers treatment with PegIFN and RBV was highly effective in naïve GT2/3 patients who were adherent to therapy. The overall per protocol analysis showed 91% SVR. However, in this real-world setting in Germany, the number of patients with low adherence or lost-to-follow up was high which resulted in poor intention-to treat SVR of just 49%. Our intention-to-treat SVR data are slightly lower compared to randomized trials where well-selected patients have been included by few well-selected study centers [11], [13], [19]. The recent HepNet REDD2/3 trial also showed a high discrepancy - 67% SVR versus 83% in the completer analysis. Also this trial was performed under real world-conditions involving 51 centers [20]. Reasons for the discrepancy between PP and ITT results are various. Treatment discontinuation due to side effects of PegIFN may be relevant, which call for IFN free therapies. However, in many cases patients were just non-compliant and did not return during or after treatment. Another major finding of our study was that treatment was unnecessarily prolonged or rather not shortened in a large portion of patients despite clear recommendations stated in national and international guidelines [15]. Only one center treated all easy to treat G2/3 patients with RVR for 16 weeks and just one fourth of centers considered RGT in the majority of patients. Importantly, over-treatment did not lead to an increase in SVR rates but may have increased the number of adverse events and certainly raised the costs of treatment dramatically. This suggests that the implementation of guidelines still needs to be improved. HCV therapy is a rapidly moving field and has seen tremendous changes in the recent years through the approval of different DAA. In some countries like Germany or the US sofosbuvir is already available and SOF/RBV ± PegIFN has become the new standard of care for most HCV GT2/3 patients and other DAA are already on the way. We think that our findings are indeed of great importance for this new era of DAA combination regimens. There cannot be a doubt that treatment of chronic hepatitis C with DAA will become highly expensive in most countries. Adherence to the exact treatment recommendations will be essential. One pill of sofosbuvir costs about 700 € in Germany or 1,000 US $ in the US (April 2014). Our data showed that only few centers followed guidelines very strictly. Current guidelines recommend 12 weeks SOF/RBV treatment for GT2 and either 24 weeks of SOF/RBV or 12 weeks of PegIFN/RBV/SOF in GT3 patients [17], [18]. If again treatment is prolonged in only some patients, costs will increase significantly, which will be an unacceptable burden for the national health care systems. Certainly, it has been considered that over-treatment with the IFN free SOF/RBV regimen may be very tempting due to very low frequency of side effects. Given the high costs of these modern therapies a dropout of more than 40% as documented in our study often due to poor compliance may not be acceptable. However, due to the better tolerability, treatment adherence may also improve. Nevertheless, the EASL recommends on-treatment HCV RNA testing at week 2 and 4 during SOF containing regimens to ensure patients adherence [18].

Finally, our data also confirmed that RGT treatment of naïve GT2/3 patients with low baseline viral load, no cirrhosis and RVR according to guidelines is possible and lead to high SVR in patients. Patients who are eligible to RGT and maintain on PegIFN/RBV therapy achieved SVR in 93%. SVR was not inferior to longer treatment of 24 weeks and saved 4,692 € per patient. This may be in particular relevant for GT3 patients who require 24 weeks of SOF/RBV. About 70% of IFN eligible and adherent GT3 patients achieved RVR with an SVR rate in>90% in our study. Similar data have been reported in randomized trials, i.e. 67% RVR with 85% SVR [19] or 71% RVR with 81%–90% SVR [13]. These SVR rates in GT3 are not inferior to SVR rates reported for 24 weeks SOF/RBV [9] or PegIFN/RBV/SOF [21] but therapy costs are about 50–100,000 € lower per patient (Table 5). As PegIFN based SOF therapy is recommended by recent guidelines, it also has to be discussed whether naïve PegIFN tolerant GT3 patients without cirrhosis and low baseline HCV RNA <800,000 IU/mL should be treated with dual PegIFN/RBV. A pre-selection based on IFNL3 polymorphism may further increase the number of patients eligible for this approach [22]. Patients without RVR can have low SVR [19] and may require an intensive treatment, which could be an add-on of SOF for 12 weeks. Recently, it has been shown that PegIFN/RBV plus SOF for 12 weeks resulted in 83% SVR in 24 difficult to treat GT3 patients from Texas [21]. An RGT approach would reduce treatment costs by more than 50,000 € per SVR in IFN eligible naïve patients compared with flat PegIFN/RBV/SOF for 12 weeks (Table 5). However, this approach has not been prospectively evaluated and four weeks longer exposure to PegIFN is required.

**Table 5:** Costs per treatment schedule in naive GT2/3. *Valence trial [9] ** Lonestar-2 trial [21].

Patients	Treatment	Duration in weeks	ITT SVR (%)	PP SVR (%)	Costs in € per treated patient	Costs per ITT SVR	Costs per PP SVR
All G2	PegIFN/RBV	24	61	96	12,750	20,902	13,281
All G3	PegIFN/RBV	24	48	90	12,750	26,563	14,167
							
G3 LVL no cirrhosis	PegIFN/RBV RGT if RVR	16 (75%)	70	92	6,375	6,830	5,197
	PegIFN/RBV/SOF RGT if no RVR	4+12 (25%)	83	83	15,070	20,633	20,633
	combined				21,445	27,463	25,830
							
All G3	Peg-IFN/RBV/SOF**	12	83	83	66,500	80,120	80,120
All G3	SOF/RBV*	24	94	94	125,000	132,979	132,979

In summary, treatment of naïve patients with genotype 2 and 3 with PegIFN/RBV is highly effective in patients who tolerate PegIFN and maintain on treatment, especially in patients with low baseline HCV RNA <800,000 IU/mL, no cirrhosis and rapid virological response. Thus this selected group of patients should still be considered for PegIFN/RBV in particular in healthcare systems with limited resources even if sofosbuvir is available and still be co-administered with PegIFN. However, our data show that the majority of patients who are eligible for shorter treatment have been treated too long, which resulted in additional unnecessary costs and possible adverse events. On the other side, many patients are lost to-follow-up or discontinue treatment, which result in an overall low intention-to-treat SVR and especially increase the cost per SVR. If unchanged, overtreatment and non-compliance will raise the price of SVR to unacceptable values if SOF or other DAA with comparable costs are used. For all treatment concepts including IFN free regimens, adherence to guidelines needs further improvement, as this will not only optimize the efficacy but also the efficiency of treatment.

## Supporting Information

File S1 **In- and exclusion criteria.**
(DOC)

## References

[1] Cornberg M, Razavi HA, Alberti A, Bernasconi E, Buti M, et al (2011) A systematic review of hepatitis C virus epidemiology in Europe, Canada and Israel. Liver Int Off J Int Assoc Study Liver 31 Suppl 2: 30–60 10.1111/j.1478-3231.2011.02539.x 21651702

[2] Mohd Hanafiah K, Groeger J, Flaxman AD, Wiersma ST (2013) Global epidemiology of hepatitis C virus infection: new estimates of age-specific antibody to HCV seroprevalence. Hepatol Baltim Md 57: 1333–1342 10.1002/hep.26141 23172780

[3] Maasoumy B, Wedemeyer H (2012) Natural history of acute and chronic hepatitis C. Best Pract Res Clin Gastroenterol. 26: 401–412 10.1016/j.bpg.2012.09.009 23199500

[4] Cornberg M, Deterding K, Manns MP (2006) Present and future therapy for hepatitis C virus. Expert Rev Anti Infect Ther 4: 781–793 10.1586/14787210.4.5.781 17140355

[5] Sarrazin C, Hézode C, Zeuzem S, Pawlotsky J-M (2012) Antiviral strategies in hepatitis C virus infection. J Hepatol 56 Suppl 1: S88–S100 10.1016/S0168-8278(12)60010-5 22300469

[6] Dusheiko G, Wedemeyer H (2012) New protease inhibitors and direct-acting antivirals for hepatitis C: interferon's long goodbye. Gut 61: 1647–1652 10.1136/gutjnl-2012-302910 22936671

[7] Lawitz E, Mangia A, Wyles D, Rodriguez-Torres M, Hassanein T, et al (2013) Sofosbuvir for previously untreated chronic hepatitis C infection. N Engl J Med 368: 1878–1887 10.1056/NEJMoa1214853 23607594

[8] Jacobson IM, Gordon SC, Kowdley KV, Yoshida EM, Rodriguez-Torres M, et al (2013) Sofosbuvir for hepatitis C genotype 2 or 3 in patients without treatment options. N Engl J Med 368: 1867–1877 10.1056/NEJMoa1214854 23607593

[9] Zeuzem S, Dusheiko G, Salupere R, Mangia A, Flisiak R, et al (2013) Sofosbuvir + Ribavirin for 12 or 24 Weeks for Patients with HCV Genotype 2 or 3: the VALENCE trial. Hepatol Baltim Md 58: 733A.

[10] The price of good health (2014) Nat Med. 20: 319 10.1038/nm.3538 24710362

[11] Hadziyannis SJ, Koskinas JS (2004) Differences in epidemiology, liver disease and treatment response among HCV genotypes. Hepatol Res Off J Jpn Soc Hepatol 29: 129–135 10.1016/j.hepres.2004.02.011 15203075

[12] Von Wagner M, Huber M, Berg T, Hinrichsen H, Rasenack J, et al (2005) Peginterferon-alpha-2a (40KD) and ribavirin for 16 or 24 weeks in patients with genotype 2 or 3 chronic hepatitis C. Gastroenterology. 129: 522–527 10.1016/j.gastro.2005.05.008 16083709

[13] Dalgard O, Bjøro K, Ring-Larsen H, Bjornsson E, Holberg-Petersen M, et al (2008) Pegylated interferon alfa and ribavirin for 14 versus 24 weeks in patients with hepatitis C virus genotype 2 or 3 and rapid virological response. Hepatol Baltim Md 47: 35–42 10.1002/hep.21975 17975791

[14] Mangia A, Santoro R, Minerva N, Ricci GL, Carretta V, et al (2005) Peginterferon alfa-2b and ribavirin for 12 vs. 24 weeks in HCV genotype 2 or 3. N Engl J Med 352: 2609–2617 10.1056/NEJMoa042608 15972867

[15] Sarrazin C, Berg T, Ross RS, Schirmacher P, Wedemeyer H, et al (2010) [Prophylaxis, diagnosis and therapy of hepatitis C virus (HCV) infection: the German guidelines on the management of HCV infection]. Z Für Gastroenterol 48: 289–351 10.1055/s-0028-1110008 20119896

[16] Castéra L, Vergniol J, Foucher J, Le Bail B, Chanteloup E, et al (2005) Prospective comparison of transient elastography, Fibrotest, APRI, and liver biopsy for the assessment of fibrosis in chronic hepatitis C. Gastroenterology. 128: 343–350. 10.1053/j.gastro.2004.11.018 15685546

[17] AASLD (2014) Recommendations for Testing, Managing, and Treating Hepatitis C. Available: http://www.hcvguidelines.org/fullreport

[18] European Association for Study of Liver (2014) EASL Clinical Practice Guidelines: management of hepatitis C virus infection. J Hepatol 60: 392–420 10.1016/j.jhep.2013.11.003 24331294

[19] Shiffman ML, Suter F, Bacon BR, Nelson D, Harley H, et al (2007) Peginterferon alfa-2a and ribavirin for 16 or 24 weeks in HCV genotype 2 or 3. N Engl J Med 357: 124–134 10.1056/NEJMoa066403 17625124

[20] Manns M, Zeuzem S, Sood A, Lurie Y, Cornberg M, et al (2011) Reduced dose and duration of peginterferon alfa-2b and weight-based ribavirin in patients with genotype 2 and 3 chronic hepatitis C. J Hepatol. 55: 554–563 10.1016/j.jhep.2010.12.024 21237227

[21] Lawitz E, Poordad F, Brainard D, Hyland RH, An D, et al. (n.d.) Sofosbuvir in Combination With PegIFN and Ribavirin for 12 Weeks Provides High SVR Rates in HCV-Infected Genotype 2 or 3 Treatment Experienced Patients with and without Compensated Cirrhosis: Results from the LONESTAR-2 Study. Hepatol Baltim Md 58: LB#4.

