# Supplementary material for: Treatment of Naïve Patients with Chronic Hepatitis C Genotypes 2 and 3 with Pegylated Interferon Alpha and Ribavirin in a Real World Setting: Relevance for the New Era of DAA
Source: PLoS One. 2014 Oct 10;9(10):e108751. doi: 10.1371/journal.pone.0108751 (PMC4193764; doi:10.1371/journal.pone.0108751)
Supplement: File S1 — In- and exclusion criteria. (DOC) [file pone.0108751.s001.doc]

**Supporting information**

*A. Inclusion criteria:*

1. Male and female patients with HCV-genotype 2/3 chronic hepatitis C documented by detectable plasma HCV RNA (> 15 IU/mL) and positivity of anti-HCV antibodies.

2. Age > 18 years

3. No history of antiviral therapy with interferon alpha or pegylated Interferon alpha and ribavirin

4. Willingness to give written informed consent and willingness to participate to and to comply with the protocol.

*B. Exclusion criteria:*

1. Women with ongoing pregnancy or breast feeding

2. Male partners of women who are pregnant

3. Positive tests at screening for anti-HAV IgM Ab, HBsAg, anti-HBc IgM Ab, HBeAg, anti-HIV, HIV-RNA

4. History or other evidence of a medical condition associated with chronic liver disease other than HCV associated (e.g., hemochromatosis, autoimmune hepatitis, alcoholic liver disease, toxin exposures).

5. History or other evidence of bleeding from esophageal varices or other conditions consistent with decompensated liver disease

6. Patients with liver cirrhosis with a lesion suspicious for hepatic malignancy on the screening

7. Absolute neutrophil count (ANC) <750 cells/mm3 at screening

8. Platelet count <50,000 cells/mm3 at screening

9. Hb <10 g/dL at screening

10. Prior Interferon alpha or ribavirin therapy

11. History of severe psychiatric disease, especially depression (ICD 10 codes F30–F33). Severe psychiatric disease is defined as treatment with an antidepressant medication or a major tranquilizer at therapeutic doses for major depression or psychosis, respectively, for at least 3 months at any previous time. Patients are excluded if any history of suicidal attempts is evident. If hospitalization for psychiatric disease, or a period of disability due to a psychiatric disease are documented, psychiatric consultation is mandatory. Patients with a mild or moderate psychiatric disease (ICD 10 codes F32.0, F32.1, F33.0, F33.1) are only allowed to be included

into the trial if a regular monitoring by a psychiatrist is performed during the trial.

12. History of a severe seizure disorder or current anticonvulsant use

13. History of immunologically mediated disease (e.g., inflammatory bowel disease, idiopathic thrombocytopenic purpura, lupus erythematosus, autoimmune hemolytic anemia, scleroderma, severe psoriasis, rheumatoid arthritis)

14. History or any other evidence of autoimmune diseases

15. History or other evidence of chronic pulmonary disease associated with functional limitation

16. History of significant cardiac disease that could be worsened by acute anemia (e.g. NYHA Functional Class III or IV, myocardial infarction, ventricular tachyarrhythmias requiring ongoing treatment, unstable angina)

17. Evidence of thyroid disease that is poorly controlled on prescribed medications.

18. Evidence of severe retinopathy (e.g. CMV retinitis, macular degeneration)

19. History of major organ transplantation with an existing functional graft

20. History or other evidence of severe illness, malignancy or any other conditions which would make the patient, in the opinion of the investigator, unsuitable for the study

21. History of any systemic anti-neoplastic or immunomodulatory treatment (including

supraphysiologic doses of steroids and radiation) 6 months prior to the first dose of study drug or the expectation that such treatment will be needed at any time during the study

22. Patients with evidence for tuberculosis

23. Drug abuse within 6 months prior to the first dose of study drug and excessive alcohol consumption. Patients on methadone/polamidone/buprenorphine programs are not excluded.

24. Any investigational drug and/or participation in another clinical study prior 6 months to the actual ongoing antiviral treatment

25. Limited contractual capability
